# Tribological and thermal stability study of nanoporous amorphous boron carbide films prepared by pulsed plasma chemical vapor deposition

**DOI:** 10.1088/1468-6996/16/3/035007

**Published:** 2015-06-02

**Authors:** Shahira Liza, Naoto Ohtake, Hiroki Akasaka, Juan M Munoz-Guijosa

**Affiliations:** 1 Department of Mechanical Sciences and Engineering, Tokyo Institute of Technology, 2-12-1O-Okayama, Meguro-ku, Tokyo, 152-8552, Japan; 2 Department of Mechanical Engineering, University of Malaya, 50603 Kuala Lumpur, Malaysia; 3 Mechanical Engineering Department, Universidad Politécnica de Madrid, C/José Gutiérrez Abascal, 2, E-28006 Madrid, Spain

**Keywords:** thin film, boron carbide film, porous surface, lubrication, tribology

## Abstract

In this work, the thermal stability and the oxidation and tribological behavior of nanoporous *a*-BC:H films are studied and compared with those in conventional diamond-like carbon (DLC) films. *a*-BC:H films were deposited by pulsed plasma chemical vapor deposition using B(CH_3_)_3_ gas as the boron source. A DLC interlayer was used to prevent the *a*-BC:H film delamination produced by oxidation. Thermal stability of *a*-BC:H films, with no delamination signs after annealing at 500 °C for 1 h, is better than that of the DLC films, which completely disappeared under the same conditions. Tribological test results indicate that the *a*-BC:H films, even with lower nanoindentation hardness than the DLC films, show an excellent boundary oil lubricated behavior, with lower friction coefficient and reduce the wear rate of counter materials than those on the DLC film. The good materials properties such as low modulus of elasticity and the formation of micropores from the original nanopores during boundary regimes explain this better performance. Results show that porous *a*-BC:H films may be an alternative for segmented DLC films in applications where severe tribological conditions and complex shapes exist, so surface patterning is unfeasible.

## 1. Introduction

Due to their potential tribological applications, segment-structured surfaces have drawn much attention in recent years [1–6]. It is well known that the patterning of tribological coatings can further enhance the wear performance of an engineering surface. A previous study by Aoki and Ohtake on segment-structured diamond like carbon (DLC) films [4] found that spaces between segments can trap wear debris, which helps to reduce abrasive wear. In addition, spaces act as oil reservoirs in lubricated regime [5]. However, increasing tribological demands of modern industries has pushed the segmented DLC film development to its limits. Furthermore, the fabrication of small segments using metal meshes as masks is difficult to apply on three-dimensional substrate shapes, and complex and costly procedures are needed [1, 4, 6]. To overcome these limitations, it may be possible to obtain a quasi-patterned surface by introducing porosity, which can have a tribological function similar to that of the segmented surfaces described above. In recent years, the tribological behavior of porous surfaces has been studied in numerous investigations. These works have demonstrated the positive influence of porosity on the tribological behavior of different ceramics [7], composites [8, 9] and metal alloys [10, 11], by trapping wear debris and acting as oil reservoirs, resulting in an improved wear resistance and lower friction coefficient.

Therefore, this work will focus on the use of nanoporous hydrogenated amorphous boron carbide (*a*-BC:H) films as an alternative way to overcome some of the stated drawbacks of segmented DLC films, also offering some advantages and improvements. DLC performance can be improved by alloying it with other elements such as boron, giving rise to a range of attractive physical and mechanical properties as a wear-resistant coating for mechanical systems [12–16]. Furthermore, the doped boron was found to improve the thermal performance of graphite [17], carbon/carbon composites [18] and other forms of carbon materials [19, 20]. The study by Zha *et al* [20] found that boron can improve the oxidation resistance of carbon porous materials, due to the formation of a B_2_O_3_ thin layer over the carbon surface at high temperature (600 °C) in air, which act as a barrier for further oxidation.

Even though *a*-BC:H films’ inherent porosity is advantageous from a tribological point of view, humidity diffusion at the pores will spread through the film thickness, finally reaching the film–substrate interface, and cause film delamination. This delamination can be unpredictable and cause early life failures. In order to prevent this delamination mechanism, we have strengthened the porous *a*-BC:H films by adding a DLC film as an interlayer.

Thus, in this study we focus our attention on understanding the pore formation, thermal stability and tribological performance of *a*-BC:H films with a DLC thin interlayer to increase humidity permeation resistance. The few studies published on this kind of film only focus on the bonding configuration in boron-containing carbon films and its mechanical properties [12–15, 21–23]. This investigation presents, in addition, an analysis of the friction and wear behavior of *a*-BC:H multi layer film coatings by reviewing the role of the pores under unlubricated and boundary oil lubricated condition. For a more comprehensive understanding, the *a*-BC:H film properties will be compared with those in the conventional DLC film.

## 2. Experimental details

### 2.1. Film deposition

**Figure 1.**
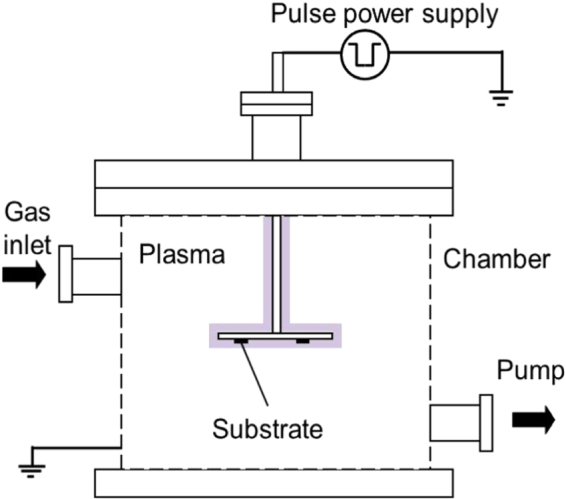
Schematic diagram of the pulsed plasma CVD system used for the film deposition.

A pulsed plasma chemical vapor deposition (CVD) system was used for the film deposition on (100) silicon substrates. Figure 1 shows a schematic diagram of the equipment. Silicon substrates were ultrasonically cleaned with distilled water, methanol and acetone for 40, 20 and 20 min, respectively. Subsequently, substrates were placed on the sample holder and loaded into the deposition chamber. DLC and *a*-BC:H films deposited on a silicon substrate do not require an interlayer. This is because they adhere very well on this kind of substrate due to silicon forming carbide during initial deposition, which is responsible for good adhesion [24]. The vacuum chamber was evacuated to a background pressure below 4.0 × 10^−7^ Pa using a turbo molecular pump. A 14 kHz monopolar pulsed power supply was used for plasma generation. The input pulsed power for plasma generation during film deposition was 2.1 W with a pulse duration of 44.2 *μ*s. Prior to deposition, substrate surfaces were sputter-cleaned by Ar plasma for 1 h at a voltage of −3 kV with a gas flow rate of 10 cm^3^ min^−1^. Table 1 shows the deposition conditions for the DLC and *a*-BC:H films. In this study, DLC interlayers were only employed between *a*-BC:H films and silicon substrates for the tribological test. The deposition conditions for the interlayer DLC film are the same as those shown in table 1 except for the deposition duration, which is reduced to 2 h.

**Table 1.** Deposition conditions of the films prepared by pulsed plasma CVD.

Deposition parameter	DLC	*a*-BC:H
C_2_H_2_ gas flow rate (cm^3^ min^−1^)	20	—
B(CH_3_)_3_ gas flow rate (cm^3^ min^−1^)	—	15
Pressure (Pa)	3	5
Bias voltage (kV)	−3	−3
Deposition time (h)	3	3
Substrate temperature (°C)	<200	<200

### 2.2. Film characterization

Film thickness was measured by means of a surface profilometer (Surftest SV-600, Mitutoyo Co., Ltd). Film chemical composition was determined by glow discharge optical emission spectroscopy (GDOES JY 5000RF, HORIBA) and x-ray photoelectron spectroscopy (XPS) system (ESCA-1700R, Physical Electronics Co., Ltd). AlK*α* x-ray source was used for XPS measurement. Film hardness and Young’s modulus were measured by indentation (PICODENTOR HM-500, Fischer Instruments K.K.). The measurements were conducted under a load of 0.8 mN for 100 different indentations. The surface of the deposited films was examined by optical and atomic force microscopy (AFM) (SPA300, SII Seiko Instruments Co., Ltd). For the pore fraction calculation, the surface area measurement was done by ImageJ free software using binarized optical microscopy images.

### 2.3. Pores stability evaluation

A local oxidation experiment was carried out in humid condition for 90 h on an *a*-BC:H film in order to study the pore stability. Boiled water and a humidity sensor were placed inside a glass container to mimic a high humidity environment, and sample was not inserted until ambient room temperature (27–29 °C) was reached. The measured relative humidity was up to 99%, and was maintained during the whole oxidation process. The oxidized film samples were examined by scanning electron microscopy (SEM) with an electron probe micro-analyzer (EPMA JXA-8200, JEOL), Fourier transform infrared (FTIR) spectroscopy (FT/IR-4200, JASCO International Co., Ltd) and x-ray diffractmeter (XRD X’Pert-Pro-MRD, Philips). For the XRD examination, the substrate was replaced from silicon to aluminum to avoid multiple embedded peaks on the spectra.

### 2.4. Thermal stability evaluation

The thermal stability of DLC and *a*-BC:H films was measured by annealing the deposited film at 500 °C under ambient conditions. Annealing duration was 1 h. The changes in the surface morphologies and chemical bonding of the annealed film were observed by optical microscope and FT/IR, respectively.

### 2.5. Tribological evaluation

In order to understand the wear and friction behavior of the deposited film, a conventional (S-DLC1) ball on disk tribometer was employed at dry and oil boundary lubricated conditions. In tribological applications, DLCs are often used as coatings for automotive parts, as well as for tools or dies. Most of these parts are made of carbon steel, which provides direct contact between DLC and steel in the tribo-system [25]. For this reason, a steel ball was chosen the counterpart in the ball on disk sliding tests. The Vickers hardness and modulus of elasticity of the steel ball are 800 HV and 210 GPa, respectively. SAE 10 W-30 engine oil and SYTOX^®^ Green nucleic acid stain fluorescent dye fluid were used as lubricants. One fluid drop was added directly onto the film surface before initiating the tests. A 6 mm diameter stainless steel ball was pressed against the film with a normal load of 1 N.

Tests were performed at a sliding speed of 0.209 ms^−1^ at ambient laboratory conditions of 25 °C and 32–56% relative humidity. The duration of each sliding test was fixed at 100 000 sliding cycles, corresponding to 250 min. A laser microscope (VK-9700 3D Laser Scanning Microscope, KEYENCE) was used to obtain the cross sectional area of the worn track. This area was used to calculate the wear volume loss. The wear rates were calculated as wear volume divided by sliding distance. The worn surfaces of the deposited films and steel balls were characterized using scanning electron microscopy (SEM, VE-8800, KEYENCE). Finally, the porosity on the worn track was observed by fluorescence microscopy (Eclipse 80*i*, NIKON).

## 3. Results and discussion

### 3.1. Film characterization

#### 3.1.1. Film composition and mechanical properties

Table 2 shows the chemical composition and properties of the deposited films prepared by pulsed plasma CVD. GDOES analysis indicated that *a*-BC:H films consist of 25.8 at.% of boron and 60.2 at.% of carbon, while DLC films consist of 84.2 at.% of carbon. The deposition rate was calculated by dividing deposition time by the film thickness.

**Table 2.** Chemical composition and properties of films prepared by pulsed plasma CVD.

	DLC	*a*-BC:H
Chemical composition (at.%):		
Boron (B)	—	25.8
Carbon (C)	84.2	60.2
Deposition rate (nm h^−1^)	367	367
Total thickness (*μ*m)	1.1	1.83
Single layer thickness (*μ*m)	—	1.1
DLC interlayer thickness (*μ*m)	—	0.73
Nano indentation hardness (GPa)	13.6	8.1
Young’s modulus (GPa)	92.5	62.2

A nanoindentation measurement was carried out on the films setting the indentation depth to be within 10% of the film thickness. As expected, results indicate that the DLC film has better mechanical properties than the *a*-BC:H film. The hardness of DLC film is 13.6 GPa and Young’s modulus of 92.5 GPa. In contrast, both hardness and Young’s modulus values for *a*-BC:H film are 8.1 and 62.2 GPa respectively. A study by Tan *et al* [26] found that hardness and Young’s modulus decrease gradually in *a*-C:B films as the boron content increase due to the lowering effect of boron on the carbon network coordination.

#### 3.1.2. Characterization of *a*-BC:H porosity

**Figure 2.**
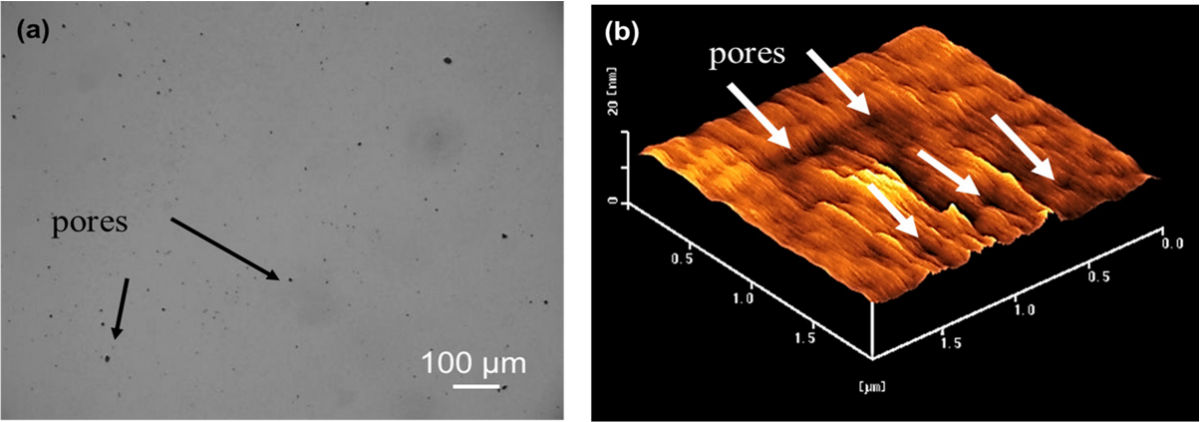
Surface topography of the *a*-BC:H film measured by optical microscopy (left) and AFM (right).

Figure 2(a) shows an optical micrograph of an as-deposited porous *a*-BC:H surface film. The pore formation on the film surface may be attributed to the oxidation process, which generally occurs during the deposition or post deposition [16, 21, 27]. The black surface area is identified as porous area on the film surface. These results were further confirmed with a surface topography measurement carried out by means of AFM, as shown in figure 2(b). Table 3 provides the fractions, diameters, depth of pores, and surface roughness of the *a*-BC:H film. The fraction of film surface containing pores was calculated using

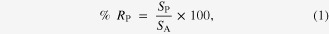
where *R*_P_ is pore per area unit rate, *S*_P_ is the pores containing surface and *S*_A_ is the total surface area. For the pore fraction calculation, the measurement of surface area was done by binarizing images as shown in figure 2(a) [28]. The black surface area is characterized as porous area on the film surface in the binary image. Pore characteristics such as diameter, height data and surface roughness are average data from the AFM image statistics. The average diameter of pores was approximately 189 ± 45 nm and average depth of pores was approximately 1.5 ± 0.7 nm.

**Table 3.** Surface characteristics of *a*-BC:H and DLC films.

Film	Pore % per area	Pore diameter (nm)	Pore depth (nm)	Surface roughness, *R*_a_ (nm)
*a*-BC:H	0.145	189 ± 45	1.5 ± 0.7	0.12 ± 0.05
DLC	—	—	—	0.33 ± 0.08

### 3.2. Pore stability

**Figure 3.**
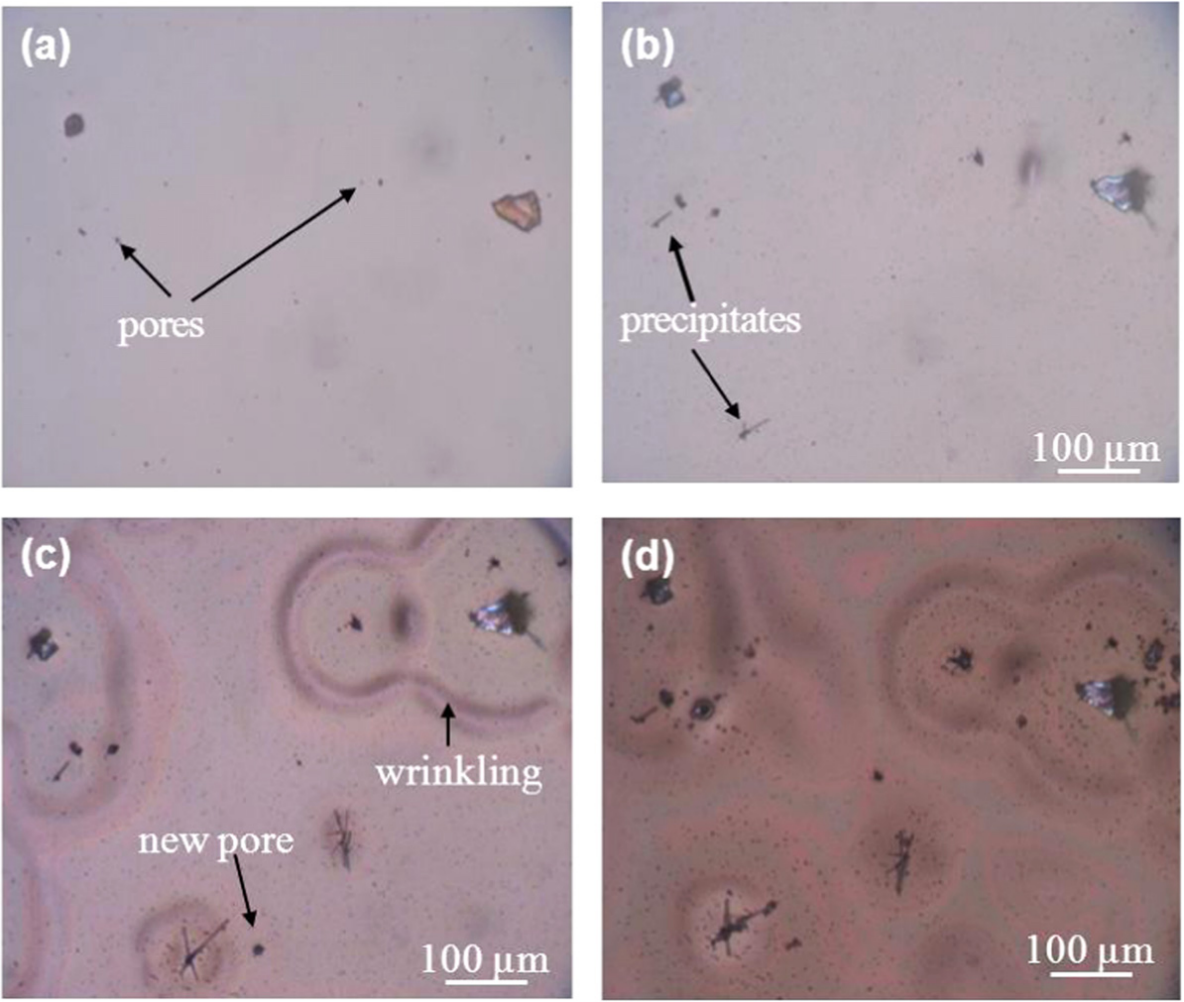
Optical micrographs show surface morphology changes on the *a*-BC:H film at (a) 0, (b) 10, (c) 45 and (d) 90 h.

Figure 3 shows the appearance of the *a*-BC:H film surface exposed to humid condition. Changes in the film surface can be observed after 10 h exposure. After 90 h, many new pores have formed, and the film surface shows wrinkles and solid precipitates around them. Optical microscope observation of the film surface reveals that the wrinkles are located at wet areas.

**Figure 4.**
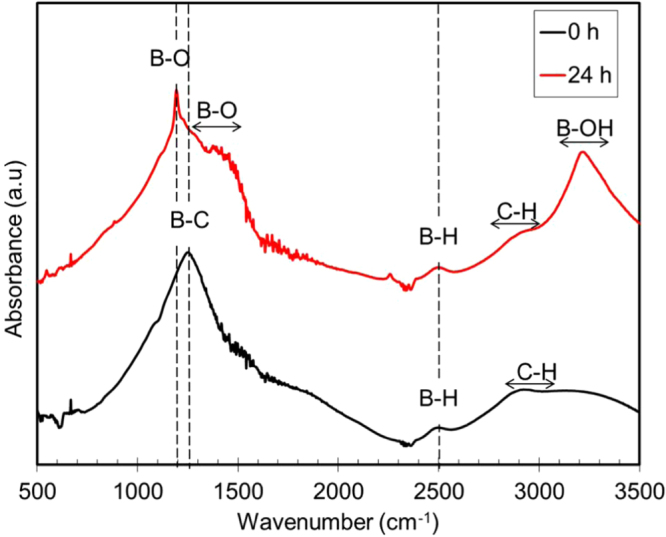
IR spectra of *a*-BC:H film 0 and 24 h after exposure to humid condition (99% relative humidity).

Further analysis by FTIR was performed to study the structural changes before and after 24 h exposure to humid conditions. Figure 4 shows the IR spectra of the *a*-BC:H film before and after the 24 h exposure. The figure clearly shows the presence of a new boron oxide (BO) peak, in comparison to the other unexposed samples. A strong absorption peak positioned at approximately at 1190 cm^−1^ (B–O deformation mode) is identified. Several weaker absorption peaks centered at 780 cm^−1^ (B–O deformation mode), 1460 cm^−1^ (B–O stretching mode), 2510 cm^−1^ (B–H stretching mode) and 3230 cm^−1^ (B–OH stretching mode) are observed [29]. The IR spectra confirmed B–O deformation mode, B–OH stretching mode and B–H stretching mode in the film exposed 24 h to the humid condition. The strong B–OH peak indicates surface adsorbed water which is diffused into the bulk of the substrate. Previous research reported that boron can act as a catalyst for the oxidation [30]. This result makes us think that the film is superficial and structural changes have been caused by the adsorbed water, which has reacted with the boron.

**Figure 5.**
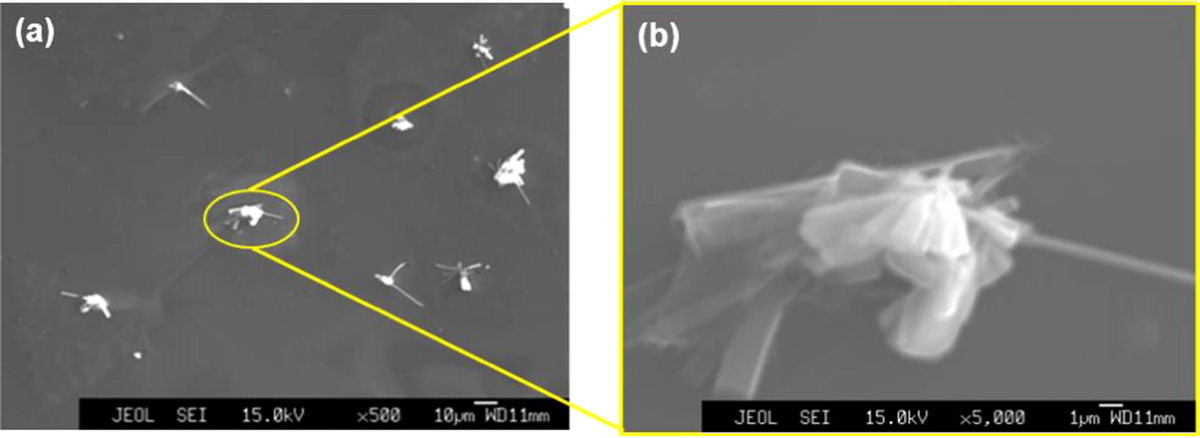
SEM images of *a*-BC:H film surface after exposure to humid condition within 24 h: (a) oxide film surface and (b) solid precipitate.

**Figure 6.**
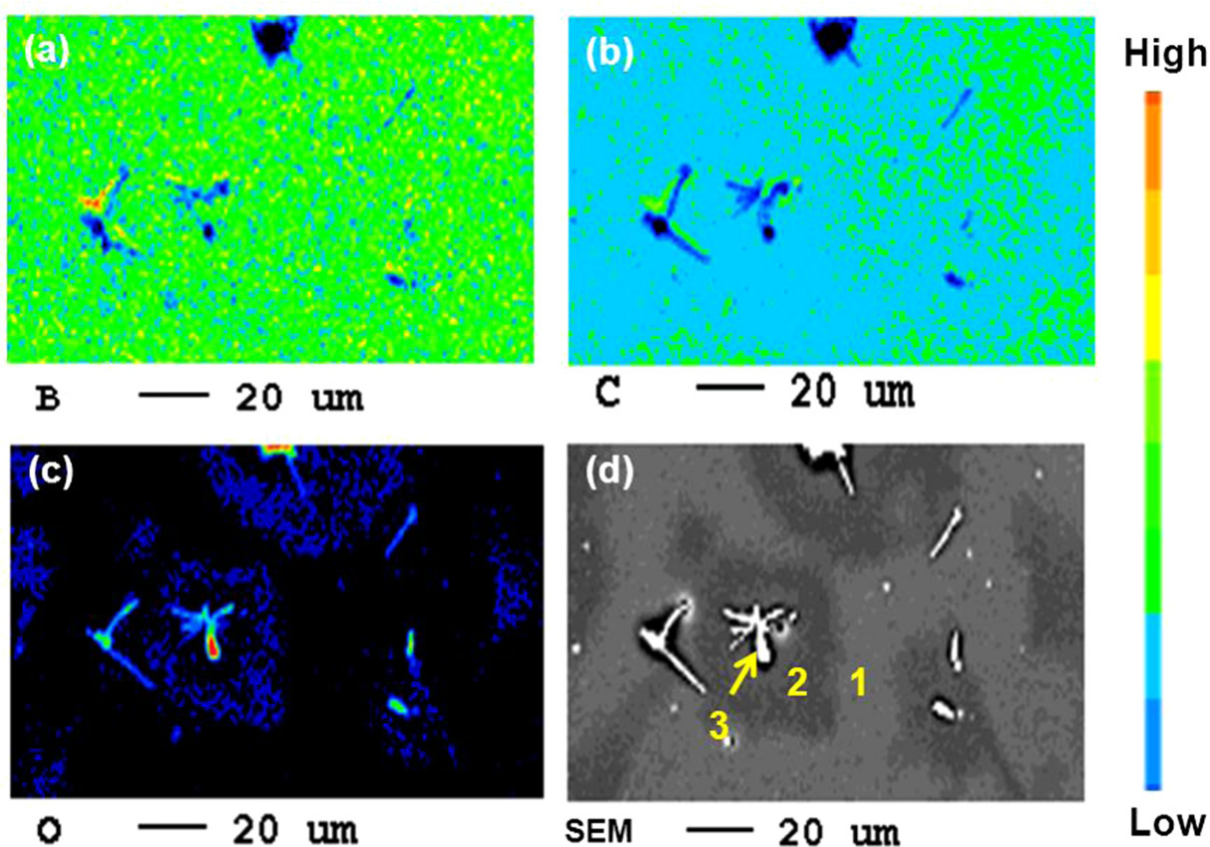
Elemental mapping of an *a*-BC:H oxide film surface after exposure to humid condition within 24 h: (a) B mapping, (b) C mapping, (c) O mapping and (d) SEM image.

Several obvious differences were also observed by SEM (figure 5). Figure 5(b) shows an enlarged image of the solid precipitates. The elemental constitution of the new pores, the surface wrinkles and the solid precipitates was studied through EPMA. The 2D EPMA elemental distribution analyses of the *a*-BC:H oxidized film and the solid precipitates are shown in figure 6. Surface wrinkles and solid precipitates lie in the oxygen rich area, which confirms the existence of the oxide film.

**Figure 7.**
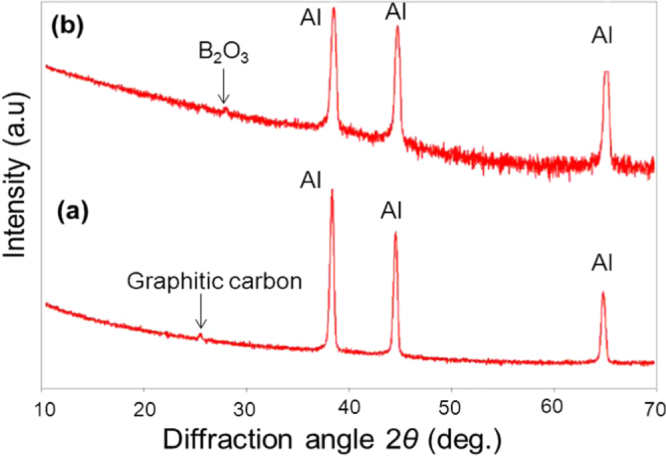
XRD patterns of the *a*-BC:H film surfaces: (a) as-deposited (b) after exposure to humid condition within 24 h.

The atomic elemental concentration in the oxide films is shown in table 4. Area no. 1 shows less oxide content in comparison to the area no. 2, corresponding area no. 2 to the pores surroundings, where wrinkles and solid precipitates are found, as can also be observed in figure 6. A higher content of O–H constituents is also found in area no. 2. Area no. 3, with the highest oxygen content, corresponds to solid precipitates. The relative atomic concentrations of B, O and C in the solid precipitates are similar to those on diboron trioxide (B_2_O_3_). This compound is known to be the product of the oxidation process [31]. The presence of B_2_O_3_ as the oxidation product in the oxide film is confirmed with the XRD analysis, as shown in figure 7. In this analysis, the Si substrate was replaced by an aluminum substrate to avoid multiple embedded peaks on the spectra. The peak corresponding to the crystalline B_2_O_3_ was observed at a diffraction angle of 2*θ* = 28° on the film surface exposed to the 24 h humid condition.

**Table 4.** Atomic concentration of B, O and C on the *a*-BC:H oxide film.

Area no.	B at. %	O at. %	C at. %
1	23.6	6.5	69.9
2	24.1	10.2	65.7
3	23.5	40.8	35.7

**Figure 8.**
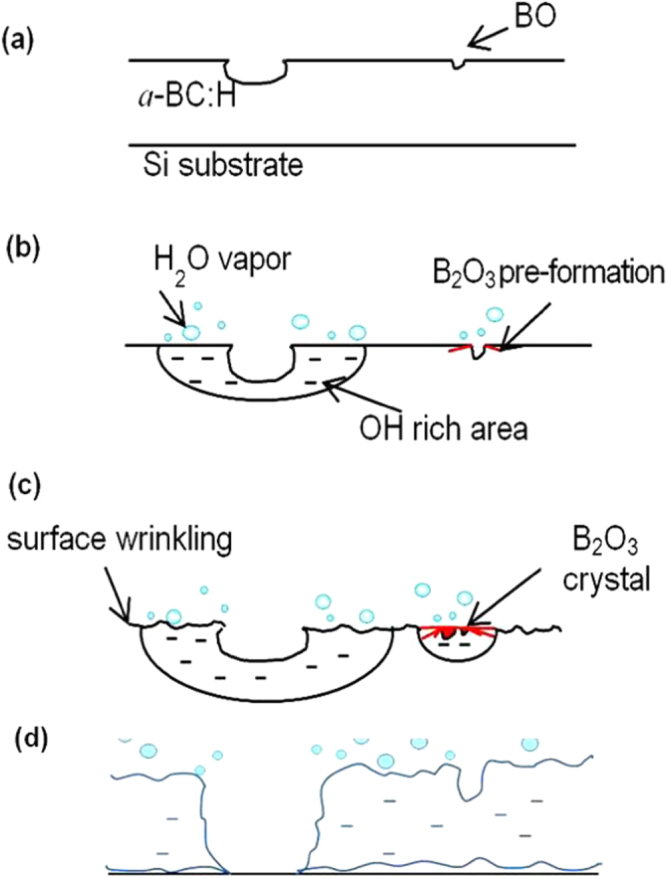
Schematic illustration showing the oxidation sequence on the *a*-BC:H oxidized film. (a) As-deposited film surface, (b) absorption of moisture, (c) diffusion into the bulk and (d) film delamination.

**Figure 9.**
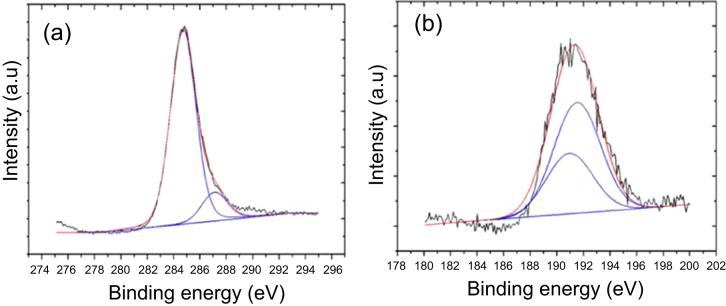
Fitted XPS spectra of as-deposited *a*-BC:H film: (a) C1s spectra and (b) B1s spectra.

As explained above, new pores, surface wrinkles in oxygen rich areas and B_2_O_3_ solid precipitates gradually appeared on the sample surface in the *a*-BC:H film local oxidation experiment. Figure 8 illustrates the oxidation sequence on the *a*-BC:H oxidized film. The oxidation process can be divided into three stages. The first one is a pre-oxidation stage, where the deposited *a*-BC:H film surface shows initial nanopores as a result of the ambient oxygen adsorption [16, 21, 27]. Figure 9 shows the bonding configuration obtained by Gaussian–Lorentzian fitting on the C1s and B1s spectral region in the XPS analysis. The B1s peak is identified at about 190.3–194.3 eV, indicating the presence of B–O bondings. This result confirms that oxygen absorption at the film surface leads to the formation of BO [32].

The second stage can be described as the adsorption of moisture at the *a*-BC:H film surface through its pores after the 10 h exposure to humid condition. Previous studies observed that pores are active sites for oxygen absorption, as its formation is originated by gaseous inclusions (O–H for example) [27, 33]. In fact, boron atoms are prone to be further oxidized and form BO at the pores, because these sites are more reactive when compared to a flat surface. As time increases, it was observed the hydro-oxygen covers almost the entire film and crystallization of B_2_O_3_ occurs at the pores. Observation near the pores shows the formation of surface wrinkles. In particular, to date surface oxidation-induced wrinkling mechanism is not clear yet. However, EPMA analysis suggests that the growth of surface wrinkles is triggered by O–H constituents. Hence, we suggest that surface wrinkling arises because of the volume increase due to water incorporation.

Later, at the last stage, the interaction between O and/or H can induce local stresses due to the oxide (B_2_O_3_) formation, leading to an increase of the compressive stress, which would in turn produce severe wrinkling, which contributes to the film delamination [33]. The present study also shows that the oxide films can act as nucleation sites for the formation of new pores, as figure 3 shows. The water reaction at the oxide film accelerates the formation of new B_2_O_3_ precipitates and pores due to the increase of OH constituents. The reaction mechanism between water and the *a*-BC:H film at room temperature can be described by equations (2) and (3)

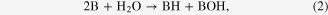


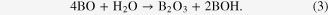


### 3.3. Thermal stability

**Figure 10.**
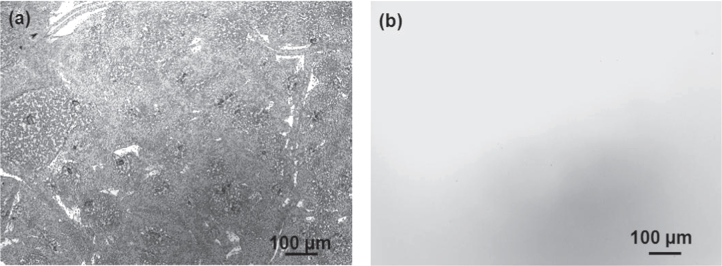
Surface morphologies of annealed film at 500 °C under ambient conditions; (a) *a*-BC:H and (b) DLC film.

**Figure 11.**
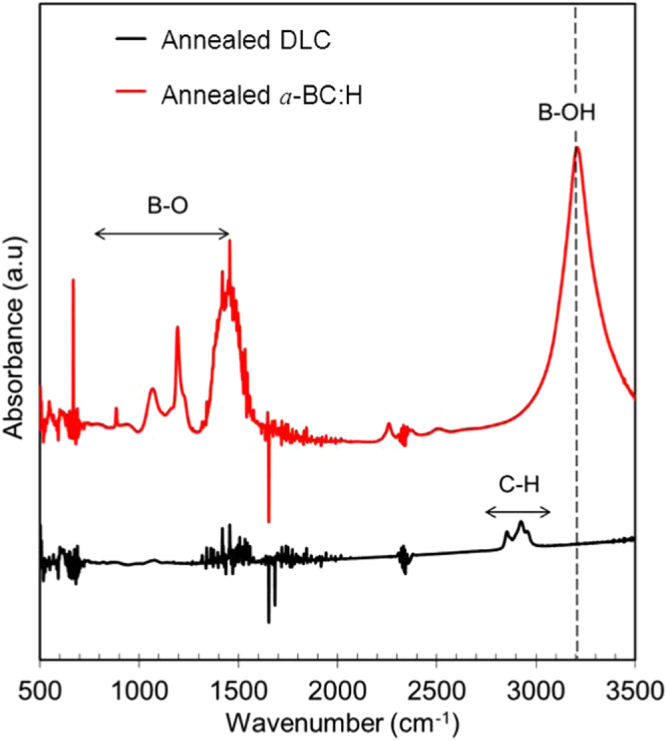
IR spectra of annealed film at 500 °C under ambient conditions.

A comparison of the thermal stability of the *a*-BC:H and DLC films was carried out by annealing both at 500 °C under ambient conditions. In order to discard any effect due to the DLC interlayer in the thermal behavior of the *a*-BC:H film, interlayer was removed for this experiment. Figure 10 shows the surface morphologies of the annealed films. This result reveals the superior thermal behavior of the *a*-BC:H film. While the DLC film was completely removed after 1 h annealing time, the *a*-BC:H film fully remained, having an oxidation layer being formed on the surface. Figure 11 shows the IR spectra of the *a*-BC:H annealed film after 1 h annealing time. The excellent thermal stability of the *a*-BC:H film at high temperature can be explained by the role of boron, which inhibits carbon oxidation by forming a B_2_O_3_ oxide layer on the film surface above 450 °C, which acts as an O_2_ diffusion barrier and an active site blocker [30, 34].

### 3.4. Tribological studies

#### 3.4.1. Friction coefficient

**Figure 12.**
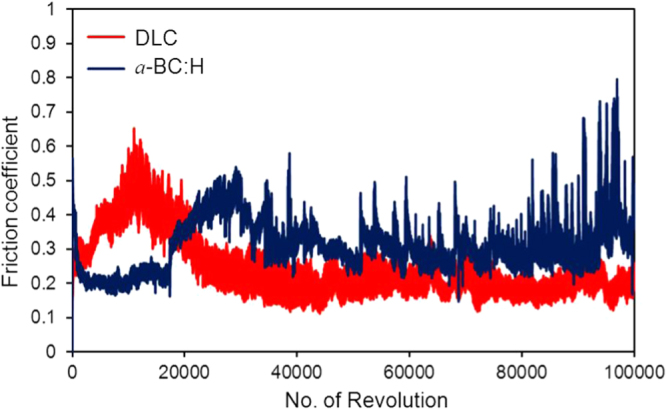
Friction coefficient in unlubricated sliding between *a*-BC:H and DLC film surfaces and steel ball after 100 000 cycles (3135 m of sliding distance).

**Figure 13.**
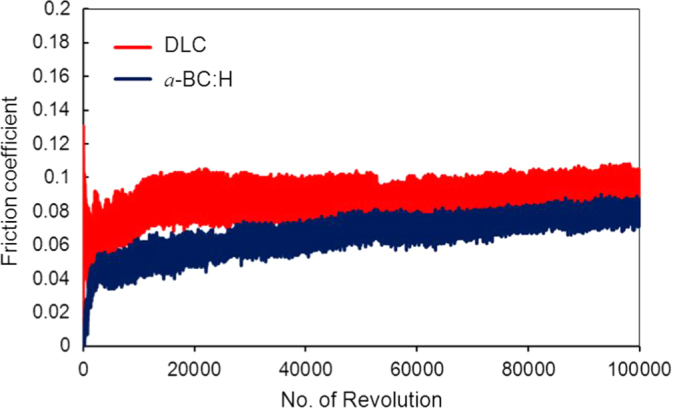
Friction coefficient in oil boundary lubricated sliding between *a*-BC:H and DLC film surfaces and steel ball after 100 000 cycles (3135 m of sliding distance).

Figures 12 and 13 show the variation of the friction coefficient (COF) of DLC and *a*-BC:H films with interlayer under unlubricated and oil boundary lubricated sliding, respectively. In the *a*-BC:H film under dry condition, the COF starts at a relative low value (0.2), increasing up to 0.35 after approximately 20 000 sliding cycles. The differences between these two values will be discussed in section 3.4.2. In contrast, the COF of the DLC film under dry condition is higher at the beginning, reaching values up to 0.6, and, after a running-in period, it has a lower steady state value (0.2), very similar to that of the *a*-BC:H film.

For oil lubricated sliding, after 100 000 sliding cycles the COF of *a*-BC:H has a lower value (0.06) than that of the DLC film (0.08). The steady increase of the boundary lubricated COF is due to the progressive reduction of the amount of oil due to the centrifugal force produced by the rotation of the substrate on the ball on disk system. Thus, friction coefficient results indicate that *a*-BC:H films with interlayer have low COF at low cycle (<20 000) in unlubricated conditions and at high cycle (100 000) in boundary oil lubricated conditions. Further analysis will be done on the worn surface of the *a*-BC:H films and steel balls to assess the factors influencing low friction in both test conditions.

#### 3.4.2. Wear behavior of *a*-BC:H films under unlubricated condition

**Figure 14.**
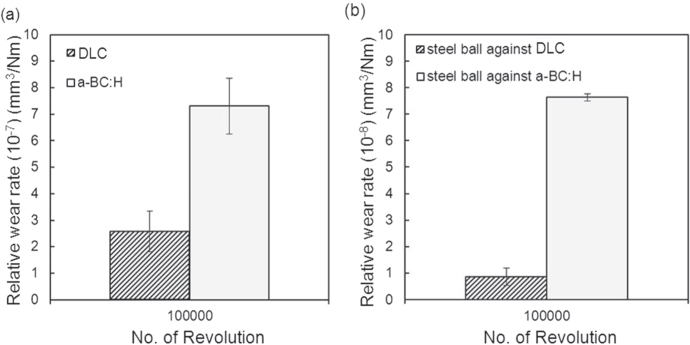
Wear rate of (a) deposited film surfaces and (b) steel ball under unlubricated condition at 100 000 sliding cycles.

The relative wear rate for both *a*-BC:H film and steel ball is higher as compared to that in the case a DLC film is used, as shown in figure 14. This expected behavior is due to the lower indentation hardness of *a*-BC:H in comparison to DLC, as described in section 3.1.1, which gives rise to a bigger material loss related to the ductile failures produced by the Hertzian nature of the contact [35].

To understand the wear behavior of the *a*-BC:H film under dry conditions, optical micrographs of the wear track on the film and steel ball surfaces are compared with those of a DLC film at different numbers of sliding cycles (see figures S1 and S2 in the supplementary information). The groove of the wear track in the *a*-BC:H worn film is very wide and deep, while the wear track of the DLC film is narrow and shallow. Similarly, the diameter of the wear scar on the steel ball sliding against *a*-BC:H film is larger than that on the steel ball sliding against the DLC film. No silicon is envisaged in any of the films, which indicates that the DLC interlayer (in the case of the *a*-BC:H film) or DLC layer are not completely removed. In addition, a transfer layer was detected on the scar surface of the ball which slides against *a*-BC:H, due to the adhesive and abrasive wear of the film [36]. The cyclic transfer layer matter addition and removal due to the sliding movement explains the instability of the measured friction coefficient at high number of cycles. On the other hand, a much smaller transfer layer is detected on the scar surface of the ball which slid against DLC. Finally, a similar abrasive wear print can be observed in both ball surfaces after 20 000 cycles, indicating that the *a*-BC:H layer may have been removed after that sliding distance. This would also explain the great wear volume difference between the steel ball sliding against *a*-BC:H and against DLC. To confirm this fact, an analysis of the wear debris characteristics is performed.

**Figure 15.**
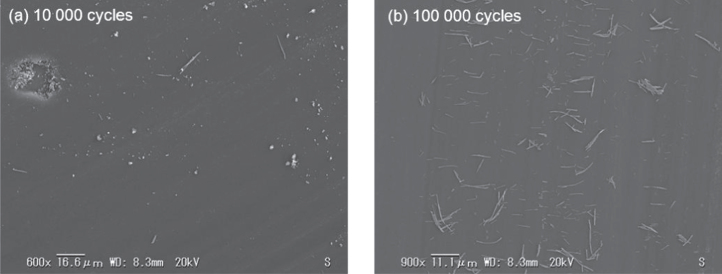
SEM images of wear scars on the *a*-BC:H film surface at (a) 10 000 and (b) 100 000 sliding cycles under unlubricated condition.

As depicted in figure 15, two different kinds of debris particles are clearly distinguished in the worn surface of the *a*-BC:H film at low and high cycles. At low sliding distance (10 000 cycles), a large amount of rounded coarse particles consisting of aggregates of different sizes and a small amount of elongated particles were found on the wear track. Micropores are also observed on the central area of the wear track (figure 15(a)). The formation of micropores is likely to occur from the prior existing nanopores, where stress concentration occurs giving rise to microcracks, which accelerate film removal [37]. In figure 15(a) it is observed that particles are being removed from the pore surroundings, which is also evidence towards the assumption of the plastic deformation origin of the newly formed pores. On the other hand, only elongated particles are found dispersed on the worn surface of the *a*-BC:H film after 100 000 sliding cycles, as shown in figure 15(b).

SEM examinations on the wear track of the *a*-BC:H film at low (<20 000) and high (100 000) sliding cycle numbers reveal two different wear behaviors, and the features of the worn surfaces were observed to change remarkably with sliding distance in unlubricated condition COF shows also different values accordingly. Under 20 000 sliding cycles, the friction coefficient of *a*-BC:H film is low (*μ*: 0.2); this result is consistent with our previous findings in sliding tests of *a*-BC:H films coated on (100) silicon substrate without any interlayer [16], where the friction coefficient was also 0.2. At low cycles (<20 000), the loose rounded coarse particles or debris are present on the sliding contact of *a*-BC:H worn surface film, which attach to the steel counter face, creating a transfer layer resulting in the low COF. The transfer layer formation may change the tribological properties significantly, as a new material pair is formed, as described by Holmberg [38]. At high (100 000) sliding cycles, elongated wear debris is found, as shown in figure 15(b). The wear debris length is between 5 and 17 *μ*m. The same debris features have also been reported by Goldsmith *et al* [39] on the sliding contact of DLC. This leads to the conclusion that after 100 000 sliding cycles film coating consists only of a DLC film layer, corresponding to a complete removal of the *a*-BC:H film layer after 20 000 cycles. The subsequent reduction of the COF in the *a*-BC:H film at 30 000 cycles, very similar to that on the DLC film at 10 000 cycles due to the formation of a DLC transfer layer at the ball scar surface. This also justifies the assumption that the *a*-BC:H has been totally removed at 20 000 cycles.

Based on the above discussion, we can conclude that material plays a basic role in the wear process of the *a*-BC:H film under unlubricated conditions. The formation of the transfer film in the *a*-BC:H-steel sliding case gives rise to a smaller COF at the early stage of sliding. Another factor that leads to the lower COF of the *a*-BC:H films in comparison with the DLC may be its lower modulus of elasticity, which increases the contact area and decreases the contact pressure between the film and the steel ball. The contact radius and pressure of *a*-BC:H and DLC were calculated using the extended Hertzian theory [40]. The contact radii for *a*-BC:H and DLC films are 71.68 and 64.78 *μ*m, respectively. While the contact pressure for the *a*-BC:H is 248.6 GPa and 303.4 MPa for the DLC film. Therefore, we suggest that *a*-BC:H films can avoid the abrasive wear that can lead to a higher COF, as that found at the early sliding stage in the DLC film. Porosity shows a little effect on the friction behavior of the *a*-BC:H film under unlubricated condition. The trapping of wear debris by pores may not play an important role in this case, since it is not easy for the pores to trap wear debris due to its small size (*d* < 1 *μ*m). However, porosity has a chief role in the wear process, as pores are crack nucleation locations. This crucial fact, which leads to the early removal of the *a*-BC:H film in dry conditions, is possitive in lubricated conditions, as will be demonstrated in section 3.4.3.

#### 3.4.3. Wear behavior of *a*-BC:H films under oil boundary lubricated conditions

In this section, the wear behavior of the *a*-BC:H film under oil lubrication using SAE 10W-30 engine oil is examined and compared with the wear in a DLC film under the same conditions. Even though the wear rates for the DLC and *a*-BC:H films are not calculated because the wear scar cross section cannot be easily measured due to oil presence in the contact surface, important conclusions can be extracted by analyzing the track and ball characteristics and correlating this information with the measured friction coefficients.

**Figure 16.**
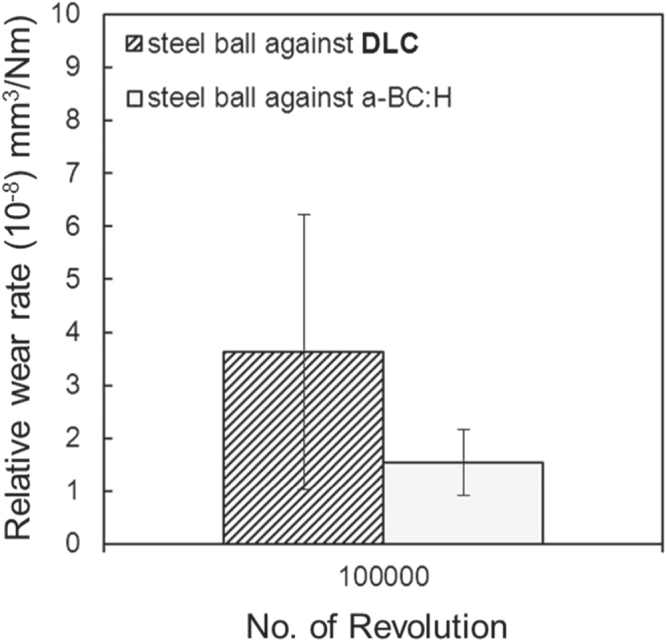
Wear rate of steel ball under oil boundary lubricated condition at 100 000 sliding cycles.

Abrasive wear can be observed in the wear scars of both balls, confirming the boundary nature of the lubricated regime. The wear rate of the steel ball sliding against the DLC film after 100 000 sliding cycles is higher in comparison with that measured in the steel ball sliding against the *a*-BC:H film, as depicted in figure 16. Up to 50 000 cycles, no transfer layer is observed in the wear scar of the steel ball sliding against the *a*-BC:H film. Figures S3 and S4 in the supplementary information show the morphology of the wear tracks of the DLC and *a*-BC:H films, respectively. Negligible wear is detected in the DLC film, and a very small wear track is observed in the *a*-BC:H film up to 100 000 cycles, producing an almost imperceptible effect on the tribological behavior, as can be observed in the friction coefficient evolution in figure 13.

These results demonstrate that porous *a*-BC:H multilayer films possess an excellent behavior under the boundary lubricated regime, being wear qualitatively comparable to that observed in DLC films and having a smaller friction coefficient. The DLC interlayer effectively prevents the delamination of the *a*-BC:H film, which occurs only after 100 000 cycles, as the transfer layer in the steel ball demonstrates, with no change in friction coefficient, which demonstrates that oil film was still present.

Based on this observation, we believe that pores have an important role in reducing friction under oil boundary lubricated conditions. Koskinen *et al* [5] have demonstrated that textured DLC film cavities at a 0.08% surface area coverage ratio, with pore diameters and depths of roughly 1–2 *μ*m and 0.5 *μ*m, respectively, can act as lubricant reservoirs. Further observations were made in order to study the role of the pores on the worn surface of the *a*-BC:H film as an oil reservoir at low sliding cycles. It is difficult to detect pores using optical and laser microscopes because we could not find a non-aggressive method to extract the remaining oil from the wear track without damaging the film. Because of this, the role of the pores as lubricant reservoirs in the tribological system is studied using a fluorescent dye as the lubricant, so that pores can be better analyzed by means of a fluorescence microscope.

#### 3.4.4. Surface pores as fluid reservoirs

**Figure 17.**
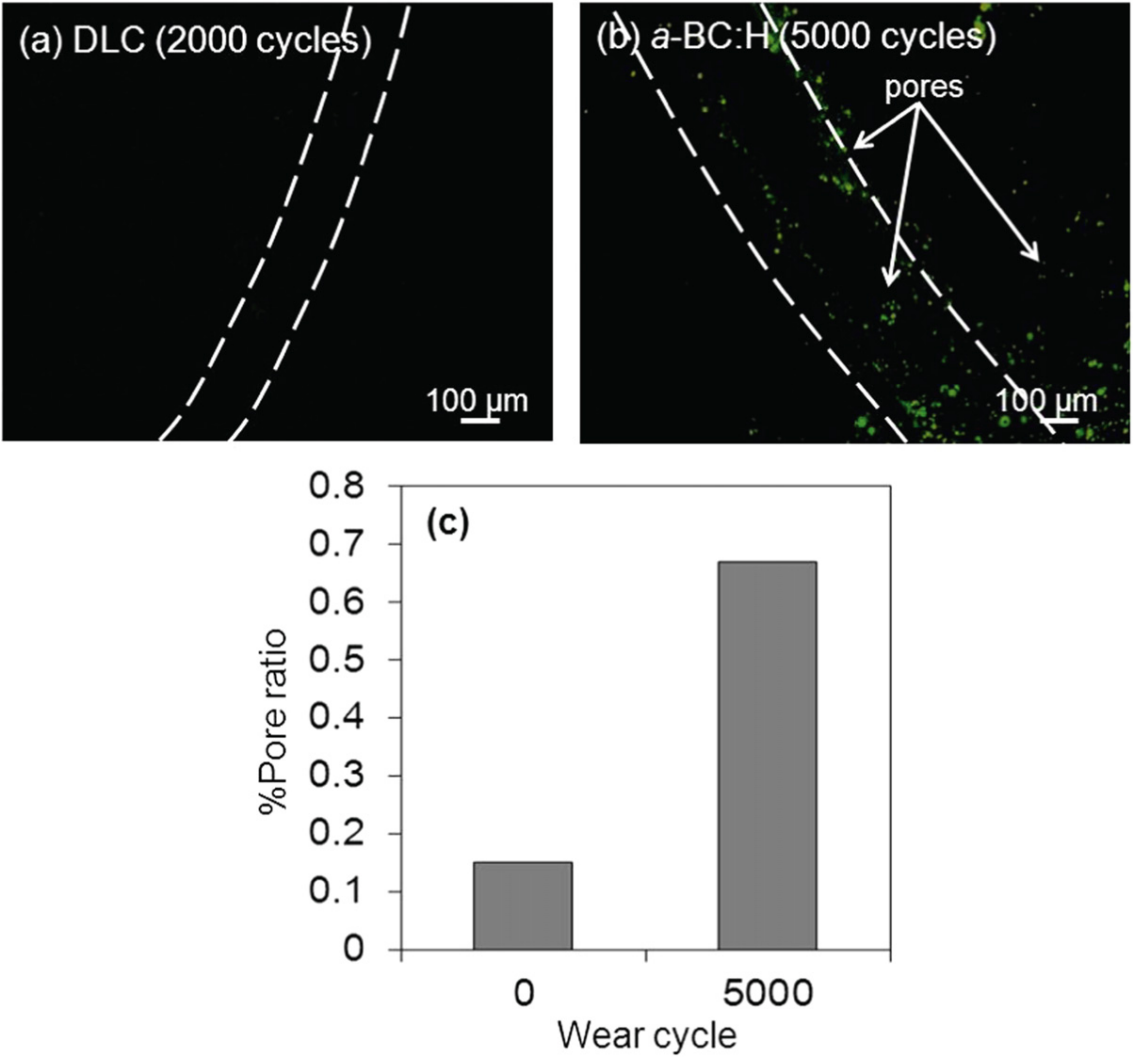
Fluorescence microscopy images of (a) DLC and (b) *a*-BC:H worn films after fluorescent boundary lubricated sliding test and (c) graph of pore ratio before and after wear test.

As mentioned above, a fluorescent dye was applied to the surface of the *a*-BC:H and DLC films in order to detect pores on the wear track during the ball on disc test. Figure 17 shows fluorescence microscopy images of the pores on the worn surface of the *a*-BC:H and DLC films and graph of pore ratio before and after the wear test. For DLC films, it is observed that film begins to delaminate after 2000 sliding cycles. Observation of the wear track of DLC and *a*-BC:H films by fluorescence microscopy revealed very few pores on the wear track of the DLC film (figure 17(a)). However, many pores (0.67% of pore ratio) were observed on the worn surface at the *a*-BC:H film (figure 17(b)). In figure 17(b), the number of pores is considerably bigger than before the test (figure 2, and direct comparison with pore distribution out of the track area). We measured the pore size for different wear test cycle ranges (for instance: 5000 and 10 000 cycles) using a fluorescent dye as lubricant. The diameter of the pores at 5000 and 10 000 cycles is 536 ± 53 and 1141 ± 520 nm, respectively. The depth of pores was found to increase from 1.6 ± 0.2 to 6 ± 4 nm at the tested cycles. In this test, we can compare not only the pore area, but also the size of the pores, which confirmed that the total and size of the pores are increased as the test cycles increased.

These findings suggest that pores on the worn surface, whose growing mechanism is similar to that occurring in dry friction conditions, can act as oil reservoirs, which prevent fluid film breaking by maintaining the lubricant supply to the tribo-contact during the sliding, resulting in low COF. In addition, the size and number of pores will be correlated to the severity of the contact conditions, so the pore growing phenomenon, which has a catastrophic effect at high intensity, as in dry friction, is beneficial in lubricated conditions. However, more investigation is needed in order to study the influence of porosity, as there are multiple variables concurrently involved, such as porosity size, shape and distribution, which affect wear and friction synergistically.

## 4. Conclusions

In order to investigate a reliable alternative for patterned DLC films in complex surfaces and severe conditions, 1.1 *μ*m thick, nanoporous *a*-BC:H films were prepared by using trimethylboron gas, B(CH_3_)_3_ by pulsed plasma CVD over a (100) silicon substrate, and its thermal, oxidation and tribological behaviors were studied and compared to those of DLC films. A DLC interlayer was used to prevent film delamination due to the diffusion and oxidation mechanisms arising at the contact of the film with ambient humidity, as the study of the humidity tests shows.

Thermal stability of the *a*-BC:H film is clearly better than that of the DLC film, without signs of delamination after annealed during 1 h at 500 °C. As expected, the nanoindentation measurement results prove that *a*-BC:H films have lower hardness and Young’s modulus than the DLC films, 8.1 and 62.2 GPa respectively. Under dry sliding tests against a steel ball, the *a*-BC:H multilayer film showed a short life, but a significant reduction in the friction coefficient compared to that in the DLC sliding, due to less pressure at contact area due to the low modulus of elasticity and early formation of a transfer film, originated as a result of the cracks nucleated at the original nanopores, which gave rise to bigger pores and quick film destruction. However, under the oil boundary lubrication regime, the friction coefficient of the *a*-BC:H multilayer film was lower than that observed in the DLC film up to a higher number of cycles (100 000). The pore growth phenomenon observed in dry friction, having less intensity, is beneficial in this case, as pores act as oil reservoirs which prevent fluid film breakage.
